# Supplementary material for: Aggregation and disaggregation features of the human proteome
Source: Mol Syst Biol. 2020 Oct 6;16(10):e9500. doi: 10.15252/msb.20209500 (PMC7538195; doi:10.15252/msb.20209500)
Supplement: Supplementary file 1 — Appendix [file MSB-16-e9500-s001.docx]

**APPENDIX**

**Table of contents**

Appendix Figure S1 - Correlation between replicates. Data from dynamic SILAC experiment with heat shock and recovery. Proteins quantified from soluble fraction (cells lysed with mild detergent).

Appendix Figure S2 - Correlation between replicates. Data from dynamic SILAC experiment with heat shock and recovery. Proteins quantified from soluble fraction (cells lysed with mild nonionic detergent; NP-40) or from samples estimating the total protein amount (cells lysed with strong ionic detergent; SDS).

Appendix Figure S3 - Characteristics of nuclear and DNA-binding proteins.

Appendix Figure S4 - Analysis of Hsp70 binding motifs.

Appendix Figure S5 - Comparison between predicted aggregation propensity and protein aggregation detected by mass spectrometry.

Appendix Figure S6 - Immunofluorescence analysis of protein localization upon heat shock.

Appendix Figure S7 - Signal to interference values in the mass spectrometry analysis.

Appendix Figure S8 - Comparison of heat-induced solubility change in pre-existing (light) and newly synthesized (heavy) protein fractions.

Appendix figure S19 - Upregulation of Hsp40s (DNAJs) upon heat shock.

Appendix Figure S10 - Correlation analysis of disaggregation slope and protein synthesis upon heat shock.

Appendix Figure S11 - Correlation between replicates in two dimensional thermal proteome profiling experiment.

Appendix Table S1 - Frequencies of aggregators and soluble proteins in different chromosomes.

APPENDIX REFERENCES


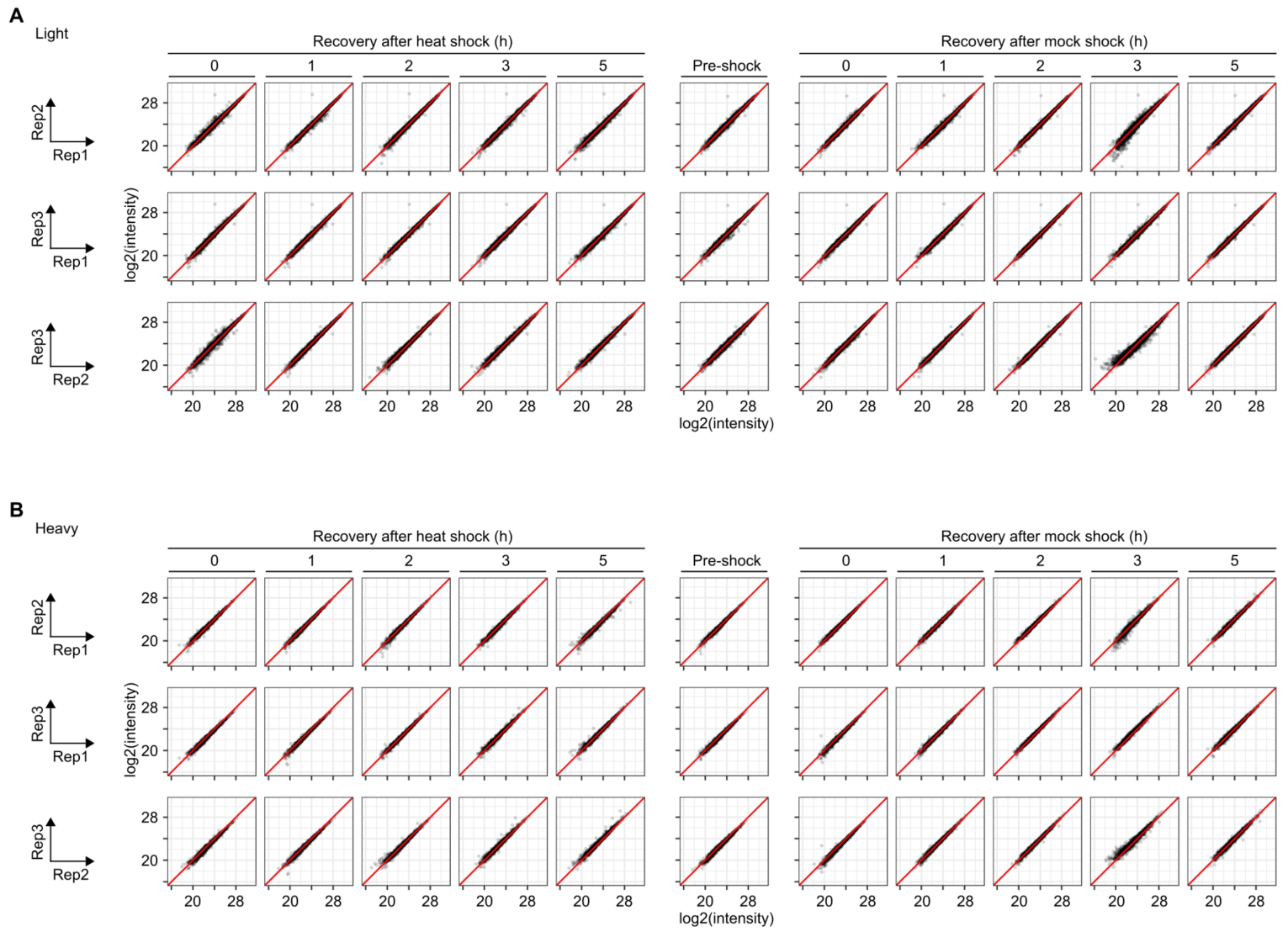


**Appendix Figure S1 - Correlation between replicates. Data from dynamic SILAC experiment with heat shock and recovery. Proteins quantified from soluble fraction (cells lysed with mild detergent).**

A-B Scatterplots showing normalized protein intensities in light (A; pre-existing proteins) and heavy (B; newly synthesized proteins) fractions.


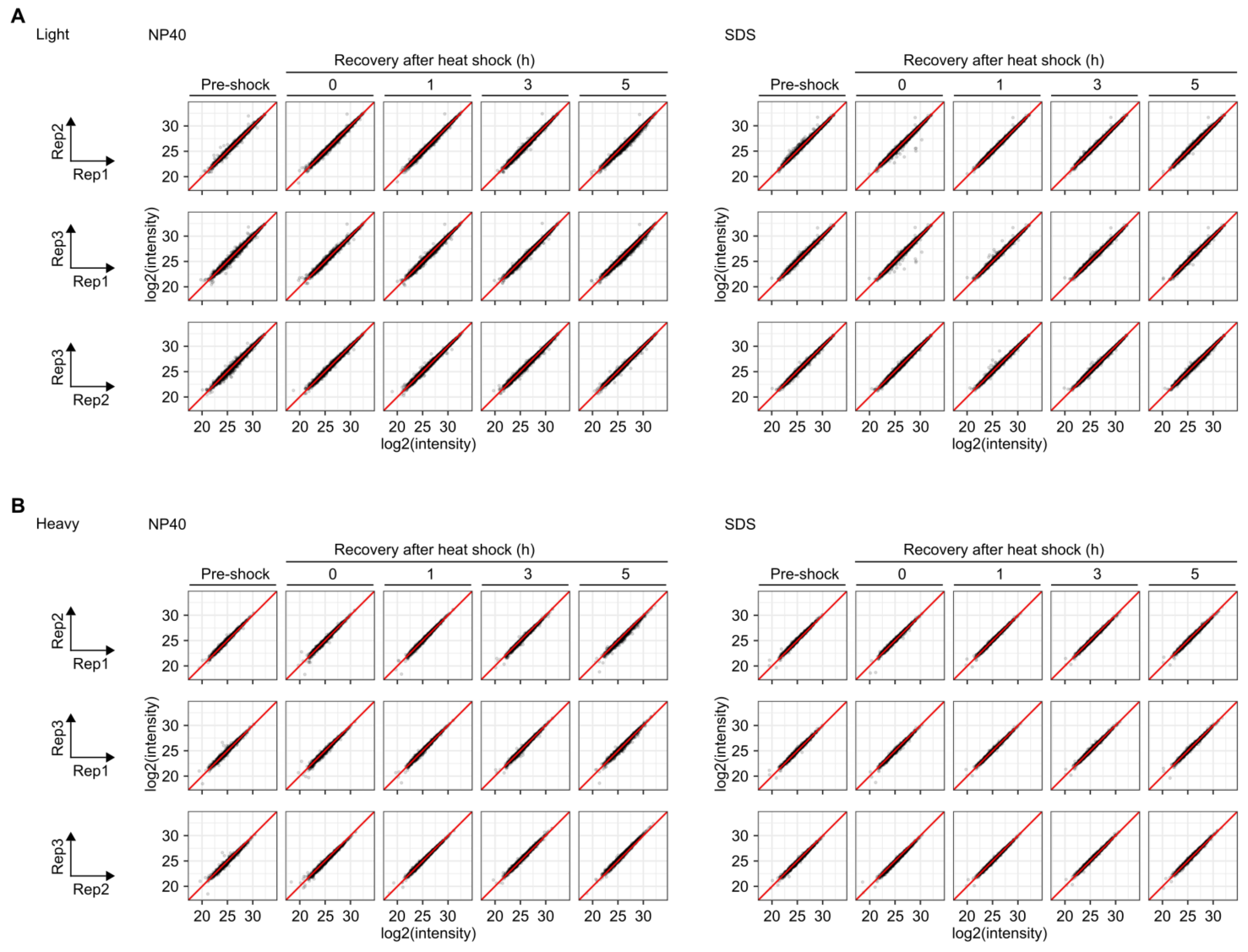


**Appendix Figure S2 - Correlation between replicates. Data from dynamic SILAC experiment with heat shock and recovery. Proteins quantified from soluble fraction (cells lysed with mild nonionic detergent; NP-40) or from samples estimating the total protein amount (cells lysed with strong ionic detergent; SDS).**

A-B Scatterplots showing normalized protein intensities in light (A; pre-existing proteins) and heavy (B; newly synthesized proteins) fractions.


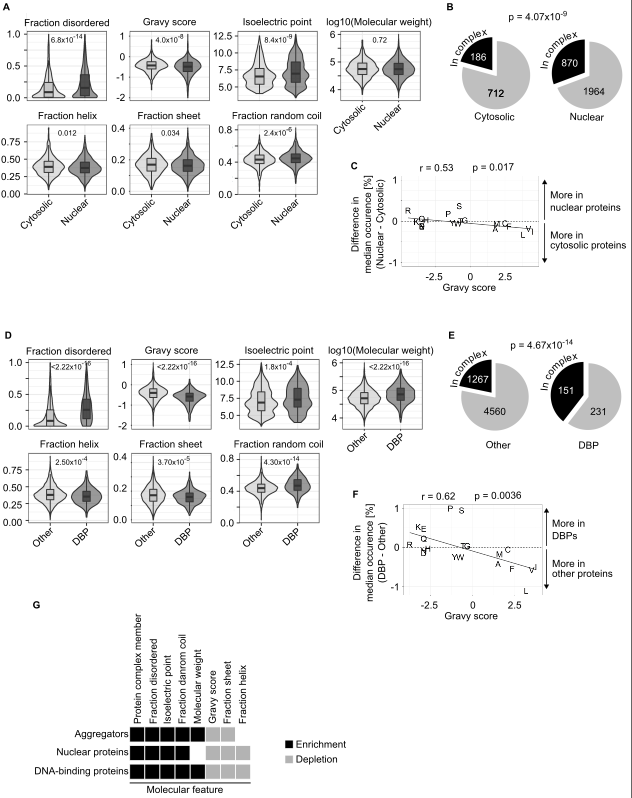


**Appendix Figure S3 - Characteristics of nuclear and DNA-binding proteins.**

A: Comparisons of structural and physicochemical features between nuclear and cytosolic proteins shown as combined violin- and boxplots (p-values are for non-parametric Wilcoxon test).

B: Protein complex members in nuclear and cytosolic proteins. Pie charts show the fraction of proteins annotated to be a member of a protein complex. The number of proteins in each segment is indicated. P-value is for Fisher’s exact test.

C: Difference in median amino acid composition between nuclear and cytosolic proteins is compared to hydrophobicity (gravy score) for each amino acid. Pearson coefficient (r) with p-value is shown for the correlation analysis.

D-F: As in A-C, expect the comparisons are between DNA-binding proteins (DBP) and all other proteins.

G: Heat map showing the enrichment (or depletion) of molecular features in aggregators (as compared to soluble proteins), DNA-binding proteins (as compared to all other binding proteins) and nuclear proteins (as compared to cytosolic proteins).

Protein was assigned as DNA-binding protein if it contained the GO term for DNA binding (GO:0003677). Protein was assigned as nuclear if it contained any of the following Human Protein Atlas annotations: ‘Nucleoli’ (GO:0005730), ‘Nucleus’ (GO:0005634), ‘Nucleoplasm’ (GO:0005654), ‘Nuclear bodies’ (GO:0016604), ‘Nuclear membrane’ (GO:0031965), ‘Nuclear speckles’ (GO:0016607), or ‘Nucleoli fibrillar center’ (GO:0001650). Protein was assigned as cytosolic if it contained the Human Protein Atlas annotation ‘Cytosol (GO:0005829)’ or ‘Cytoplasmic bodies (GO:0036464)’. The data is shown for all identified proteins in the MS analysis.


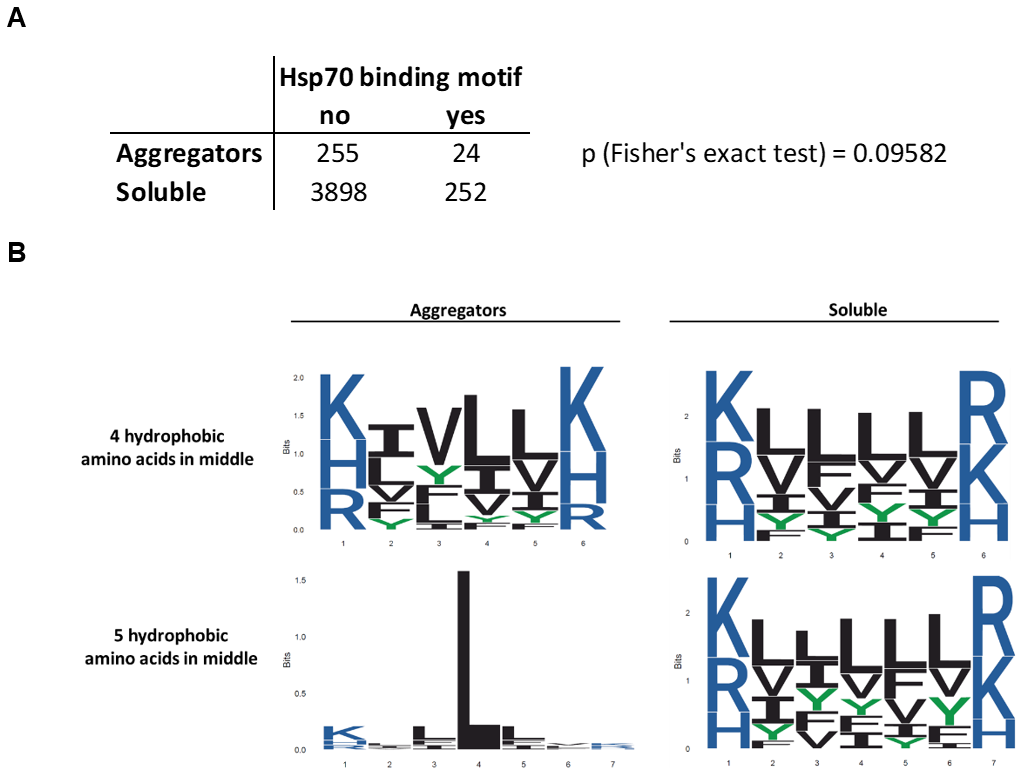


**Appendix Figure S4 -** **Analysis of Hsp70 binding motifs.**

A Frequencies of Hsp70 binding motifs identified in aggregators and soluble proteins.

B Sequence logos of the two different Hsp70 binding motifs (four or five amino acids in the middle) found in aggregators and soluble proteins.

The Hsp70 binding motif consists of four or five hydrophobic amino acids flanked by positively charged residues (Rüdiger et al, 1997).


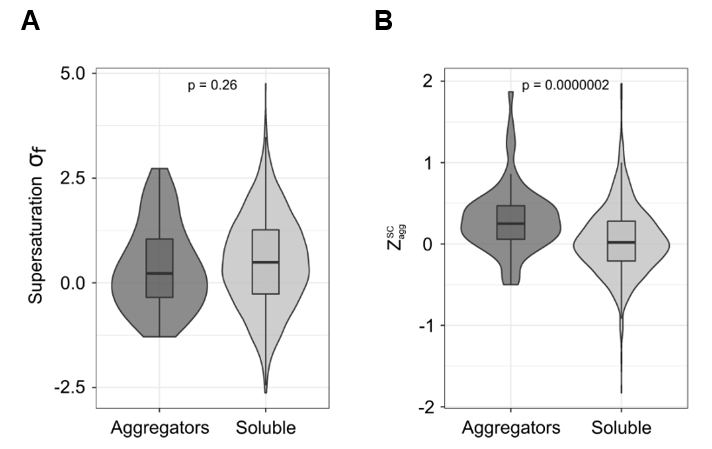


**Appendix Figure S5 - Comparison between predicted aggregation propensity and protein aggregation detected by mass spectrometry.**

Comparison of supersaturation score (Ciryam et al, 2013) (A) and structurally corrected aggregation propensity score (Z_agg_^SC^) (Ciryam et al, 2013) (B) between aggregators and soluble proteins. P-values are shown for non-parametric Wilcoxon test. Boxplots indicate median, first and third quartiles with whiskers extended to 1.5 times the interquartile range out from each quartile. Violin plots show the data distribution.

**Appendix Figure S6 - Immunofluorescence analysis of protein localization upon heat shock.**

Cells after exposure to heat shock (at 44°C) for 0, 10, 20 ,60 or 120 minutes.

After each indicated treatment, HeLa cells were fixed and annotated target proteins (green) were visualized by antibody staining. DNA was visualized by Hoechst staining (blue).

**
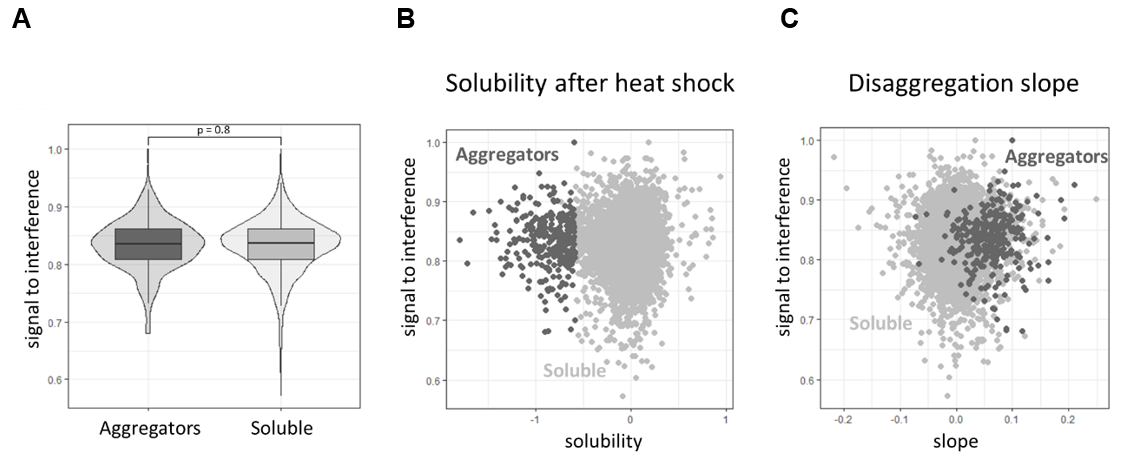
**

**Appendix Figure S7 - Signal to interference values in the mass spectrometry analysis.**

A Signal to interference values of aggregators and soluble proteins. P-value is shown for non-parametric Wilcoxon test. Boxplots indicate median, first and third quartiles with whiskers extended to 1.5 times the interquartile range out from each quartile. Violin plots show the data distribution.

B Scatterplot comparing heat-induced solubility change and signal to interference.

C Scatterplot comparing disaggregation slopes and signal to interference.

Mean peptide-level signal to interference is shown for each protein.

**
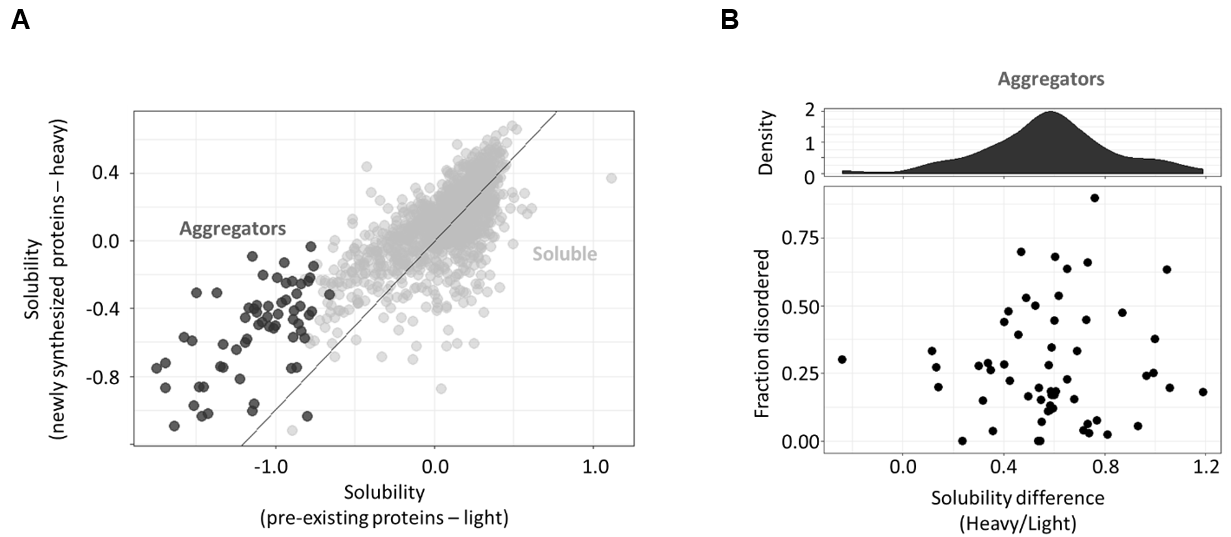
**

**Appendix Figure S8 - Comparison of heat-induced solubility change in pre-existing (light) and newly synthesized (heavy) protein fractions.**

A Scatterplot comparing the heat shock-induced solubility change in pre-existing (light) and newly synthesized heavy) protein fraction. Aggregators are highlighted with darker colour.

B Scatterplot comparing the solubility difference in pre-existing and newly synthesized protein fraction with fraction of predicted intrinsically disordered regions. The distribution of solubility difference is shown as density plot on top.

**
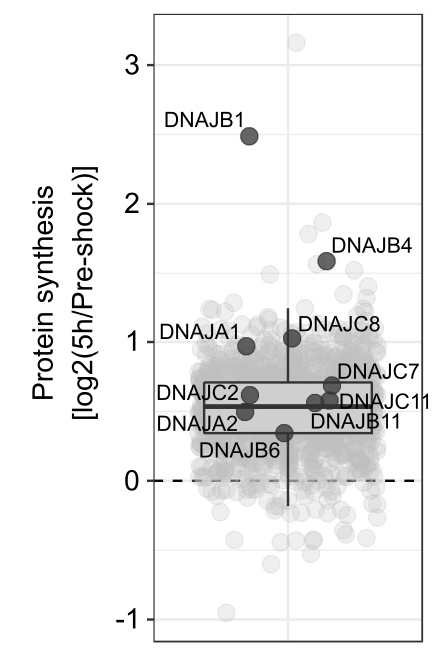
**

**Appendix figure S9 - Upregulation of Hsp40s (DNAJs) upon heat shock.**

Intensity of newly synthesized proteins at five hours after recovery from heat shock. Hsp40s (DNAJs) are highlighted and annotated.

**
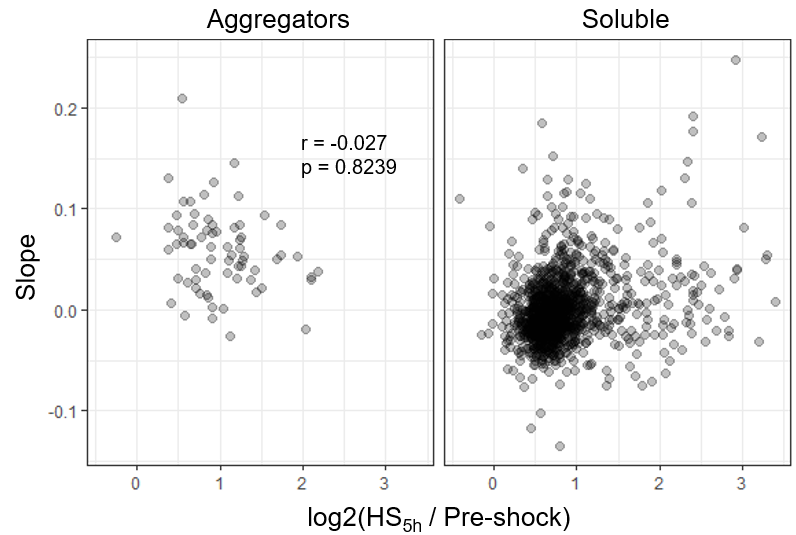
**

**Appendix Figure S10 - Correlation analysis of disaggregation slope and protein synthesis upon heat shock.**

Scatter plots comparing protein synthesis (log2-transformed ratio between five hours after heat shock and pre-shock control) and disaggregation slope. Correlation coefficient (r) with p-value shown for Spearman’s rank-order correlation.


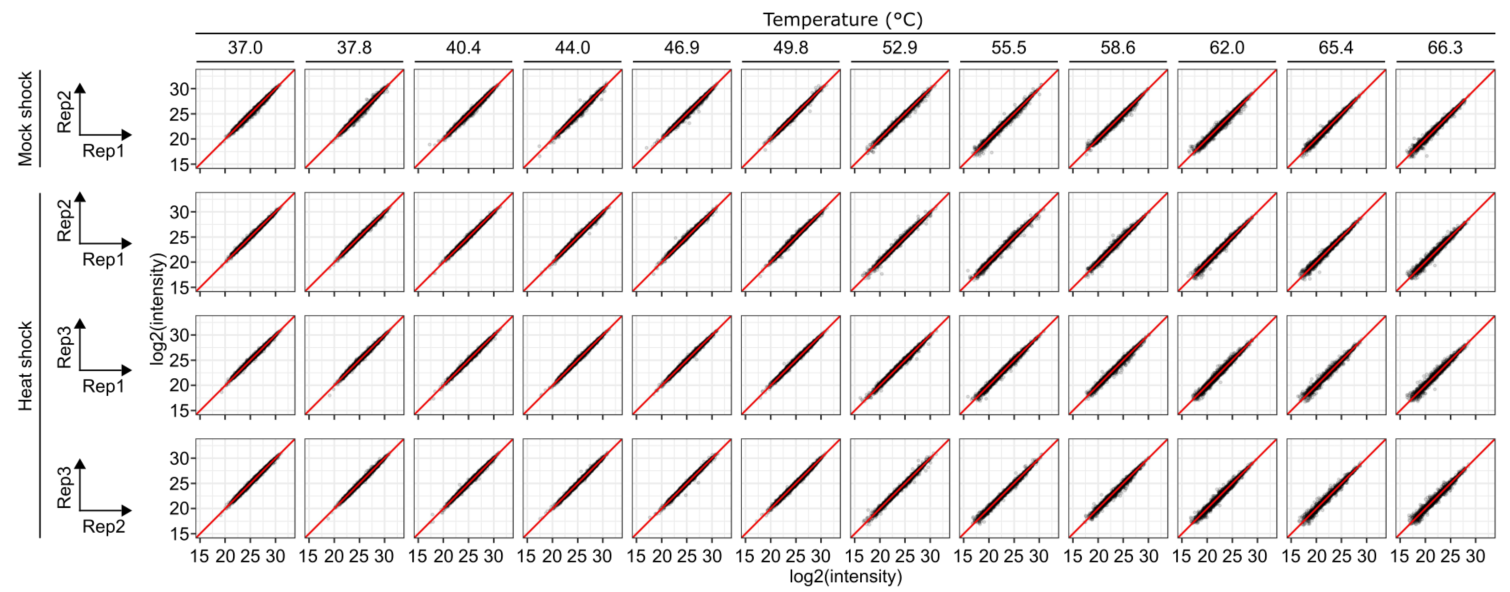


**Appendix Figure S11 - Correlation between replicates in two dimensional thermal proteome profiling experiment.**

Scatterplots showing normalized protein intensities in each condition.

**Appendix Table S1 - Frequencies of aggregators and soluble proteins in different chromosomes.**

| **Chromosome** | **Aggregators** | **Soluble** | **p-value*** | **Adjusted**  **p-value#** |
| --- | --- | --- | --- | --- |
| **1** | 16 | 223 | 0.517 | **1.000** |
| **2** | 13 | 139 | 0.184 | **1.000** |
| **3** | 8 | 125 | 0.597 | **1.000** |
| **4** | 4 | 71 | 0.614 | **1.000** |
| **5** | 7 | 99 | 0.471 | **1.000** |
| **6** | 8 | 104 | 0.390 | **1.000** |
| **7** | 11 | 112 | 0.157 | **1.000** |
| **8** | 4 | 73 | 0.638 | **1.000** |
| **9** | 4 | 65 | 0.539 | **1.000** |
| **10** | 4 | 77 | 0.682 | **1.000** |
| **11** | 9 | 122 | 0.442 | **1.000** |
| **12** | 9 | 111 | 0.337 | **1.000** |
| **13** | 1 | 38 | 0.770 | **1.000** |
| **14** | 7 | 70 | 0.173 | **1.000** |
| **15** | 4 | 70 | 0.602 | **1.000** |
| **16** | 2 | 93 | 0.967 | **1.000** |
| **17** | 10 | 130 | 0.397 | **1.000** |
| **18** | 2 | 27 | 0.334 | **1.000** |
| **19** | 12 | 125 | 0.168 | **1.000** |
| **20** | 6 | 57 | 0.153 | **1.000** |
| **21** | 3 | 14 | 0.028 | **0.667** |
| **22** | 6 | 48 | 0.083 | **1.000** |
| **X** | 7 | 82 | 0.289 | **1.000** |

* P-values were obtained using a Monte Carlo sampling approach (B = 1000) using a binomial distribution with probabilities obtained from the overall frequencies of soluble and aggregating proteins. It was then tested whether the numbers of observed soluble and aggregating proteins from each individual chromosome were significantly different from the overall trend by comparison to the sampling results.

# P-values are Benjamini-Hochberg adjusted for multiple hypothesis testing.

**APPENDIX REFERENCES**

Ciryam P, Tartaglia GG, Morimoto RI, Dobson CM, Vendruscolo M (2013) Widespread aggregation and neurodegenerative diseases are associated with supersaturated proteins. *Cell reports* 5: 781-90

Rüdiger S, Germeroth L, Schneider-Mergener J, Bukau B (1997) Substrate specificity of the DnaK chaperone determined by screening cellulose-bound peptide libraries. *Embo j* 16: 1501-7
