# Supplementary material for: Aggregation and disaggregation features of the human proteome
Source: Mol Syst Biol. 2020 Oct 6;16(10):e9500. doi: 10.15252/msb.20209500 (PMC7538195; doi:10.15252/msb.20209500)
Supplement: Supplementary file 2 — Expanded View Figures PDF [file MSB-16-e9500-s002.pdf]

Expanded View Figures

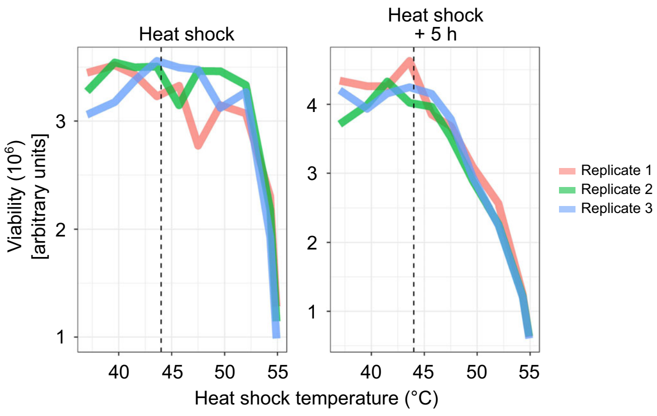

Figure EV1. Cell viability after heat shocks with different temperatures and during recovery.

Cells were exposed to a heat shock for 10 min at different temperatures (37–55°C). Cell viability (based on ATP levels) was measured right after the heat shock and after 5 h of recovery at 37°C. Dashed vertical line indicates 44°C. Data shown for three technical replicates.

Source data are available online for this figure.

© 2020 The Authors

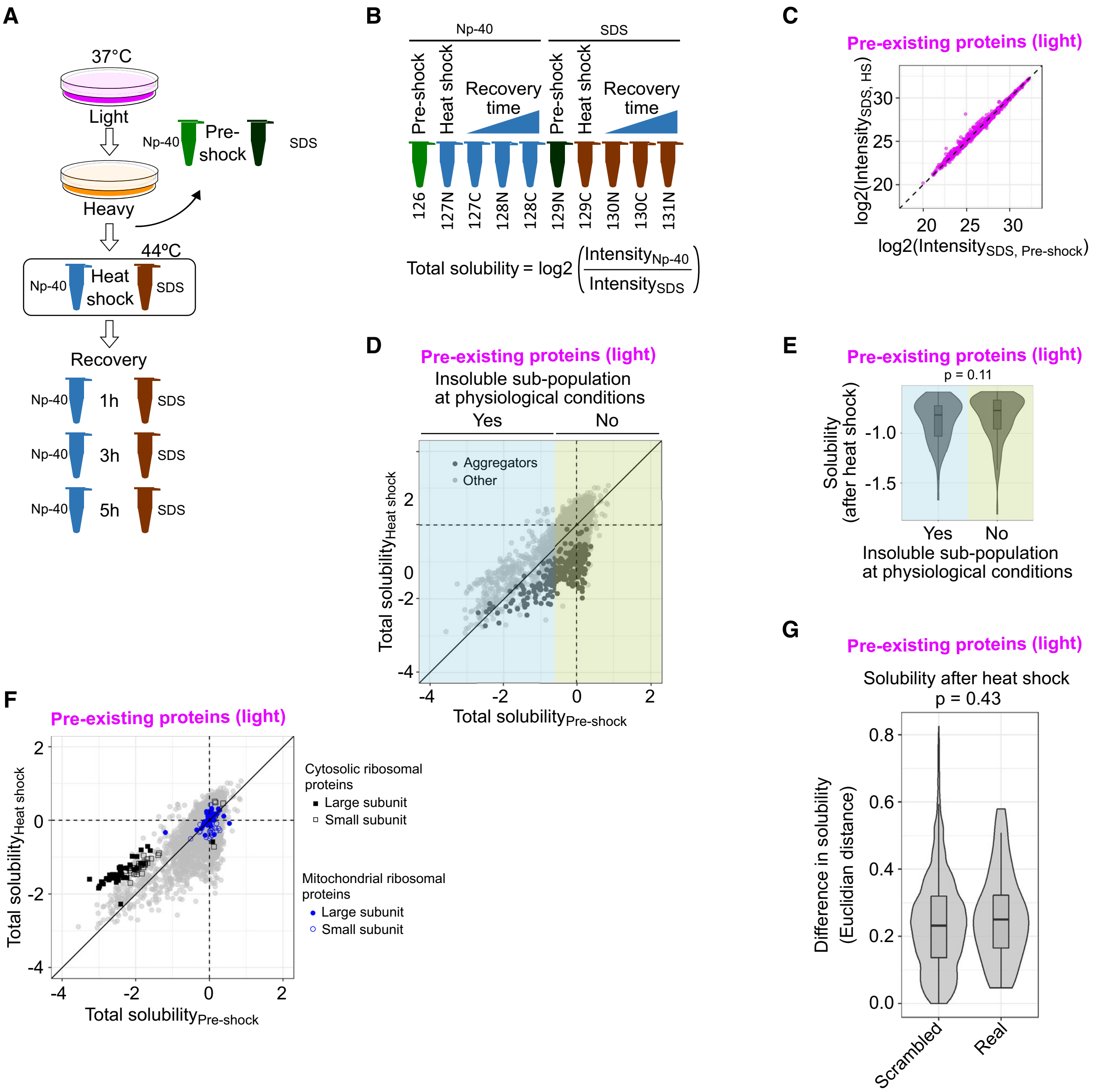

Figure EV2.

© 2020 The Authors

◀ **Figure EV2. Protein abundance, total solubility, and aggregation upon heat shock.**

A Experimental design for measuring total protein abundance and total solubility.

B Sample labeling scheme. Total solubility is calculated as log_2-transformed ratio between protein intensity in the soluble fraction (NP-40 lysis) and total protein abundance (SDS lysis).

C Scatterplot showing total protein abundance (intensity in SDS-lysed samples) of pre-existing proteins (light) after heat shock compared with pre-shock control. Medians of normalized intensities (log_2-transformed).

D Total solubility before and after heat shock. Scatterplot comparing total solubility in pre-shocked control and heat-shocked sample. Aggregators (as defined in Fig 2A) are highlighted with darker color. In addition, proteins were assigned to contain an insoluble sub-population at physiological conditions if the total solubility of the pre-shocked sample was lower than −0.6 (highlighted with cyan at the left of the figure).

E Solubility after heat shock (see Fig 2A) of aggregators with or without an insoluble sub-population at physiological conditions. *P*-value is for non-parametric Wilcoxon test. Boxplots indicate median, first and third quartiles with whiskers extended to 1.5 times the interquartile range out from each quartile. Violin plots show the data distribution. Data from at least two biological replicates.

F Total solubility before and after heat shock of ribosomal proteins. As in (D), except cytosolic and mitochondrial ribosomal proteins from large and small subunit are highlighted.

G Difference in solubility change after heat shock for aggregators in protein complexes ("Real") compared with the same aggregators randomly distributed to complexes ("Scrambled"). The difference in aggregation within each complex is estimated by calculating mean of all Euclidian distances of solubility (see Fig 2A) between aggregators. *P*-values are shown for non-parametric Wilcoxon test. The analysis includes 32 protein complexes ("Real") with at least 75% of members with good quality solubility data and include at least two aggregators. For the scrambled complex set, 10,000 complexes were created by randomly assigning aggregators from the 32 annotated complexes. The frequency distribution of aggregators in complexes was maintained in the scrambled set. Boxplots indicate median, first, and third quartiles with whiskers extended to 1.5 times the interquartile range out from each quartile. Violin plots show the data distribution. Solubility data used in the analysis are from at least two biological replicates.

Source data are available online for this figure.

© 2020 The Authors

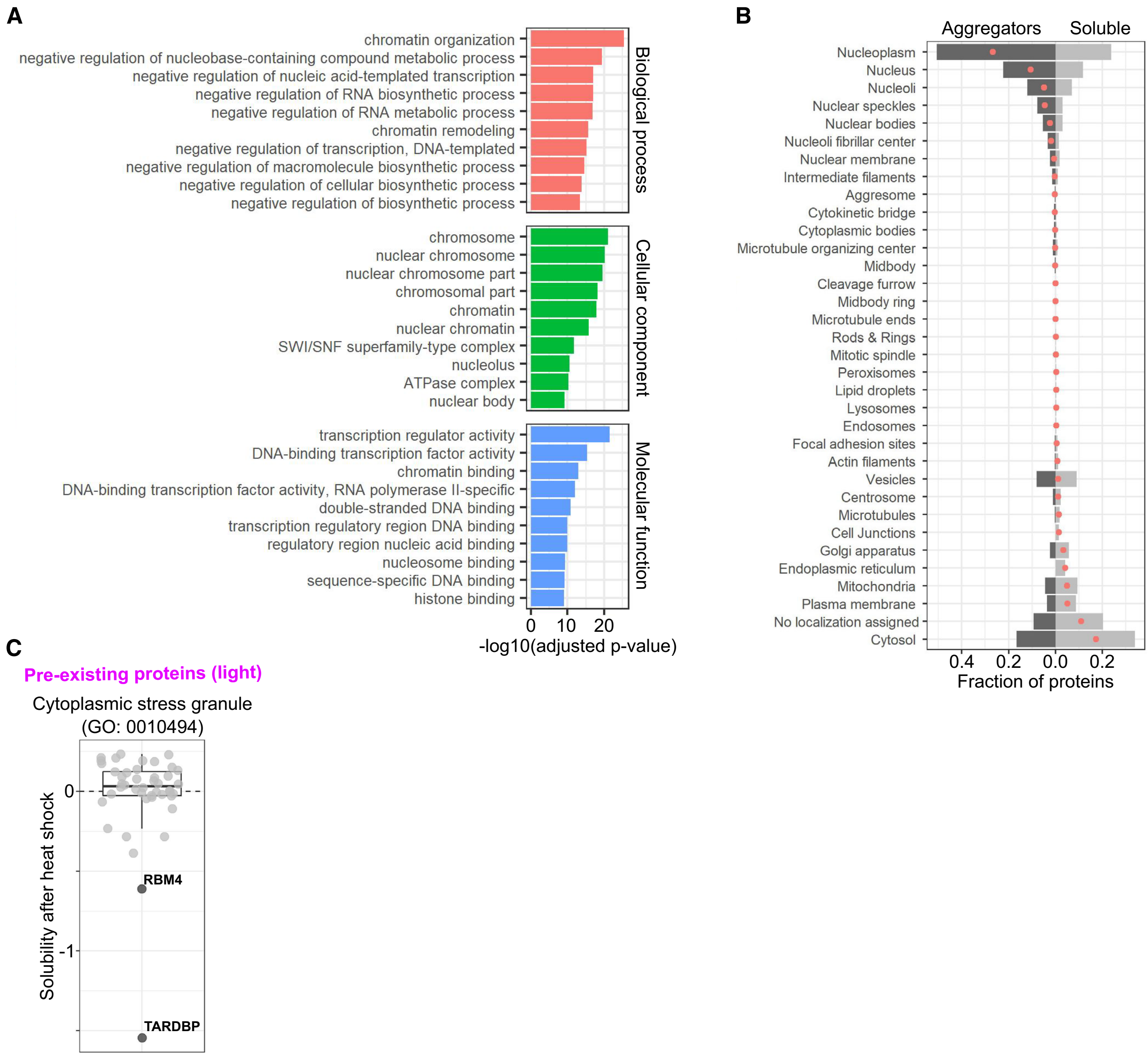

Figure EV3. Functions and localizations of aggregators and heat shock-induced solubility changes of stress granule proteins.

A Gene ontology (GO) enrichment of aggregators. Bar plot showing 10 most enriched (lowest Benjamini–Hochberg-adjusted *P*-value in hypergeometric test) terms from each GO domain.

B Localization annotations for aggregators and soluble proteins. Bar plot showing the fraction of aggregating or soluble proteins having particular localization annotation. Red dots indicate the difference between aggregators and soluble proteins.

C Solubility changes of stress granule proteins after heat shock. Heat shock-induced solubility changes of proteins with a GO term "cytoplasmic stress granule" (GO:0010494). Aggregators are labeled and highlighted with darker color.

Source data are available online for this figure.

 © 2020 The Authors

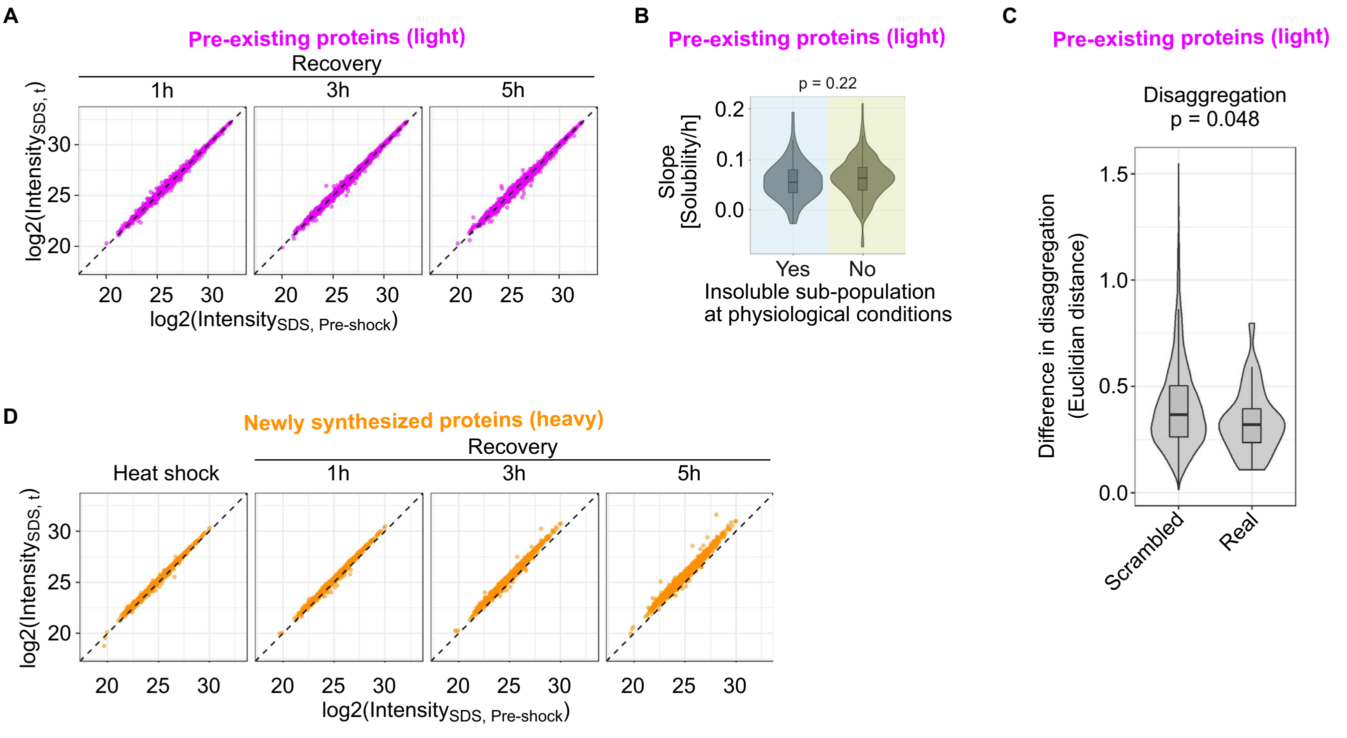

Figure EV4. Disaggregation and protein synthesis during recovery from heat shock.

A Total protein abundances of pre-existing proteins (light) during recovery from heat shock. Scatterplot showing medians of normalized intensities (log_2-transformed) of heat-shocked samples compared with pre-shocked control.

B Disaggregation slopes of aggregators with or without an insoluble sub-population at physiological conditions. *P*-value is for non-parametric Wilcoxon test. Boxplots indicate median, first, and third quartiles with whiskers extended to 1.5 times the interquartile range out from each quartile. Violin plots show the data distribution. Data from at least two biological replicates.

C Difference in disaggregation for aggregators in protein complexes ("Real") compared with the same aggregators randomly distributed to complexes ("Scrambled"). The difference in disaggregation within each complex is estimated by calculating mean of all Euclidian distances of solubility (see Fig 2A) between aggregators in each time point. To examine only the disaggregation, solubility in each recovery time point is normalized to the initial loss of solubility after heat shock prior to the distance calculation. *P*-values are shown for non-parametric Wilcoxon test. The analysis includes 32 protein complexes ("Real") with at least 75% of members with good quality solubility data and include at least two aggregators. For the scrambled complex set, 10,000 complexes were created by randomly assigning aggregators from the 32 annotated complexes. The frequency distribution of aggregators in complexes was maintained in the scrambled set. Boxplots indicate median, first, and third quartiles with whiskers extended to 1.5 times the interquartile range out from each quartile. Violin plots show the data distribution. Solubility data used in the analysis are from at least two biological replicates.

D Total protein abundances of newly synthesized proteins (heavy) during recovery from heat shock. Scatterplot showing medians of normalized intensities (log_2-transformed) of heat-shocked samples compared with pre-shocked control.

Source data are available online for this figure.

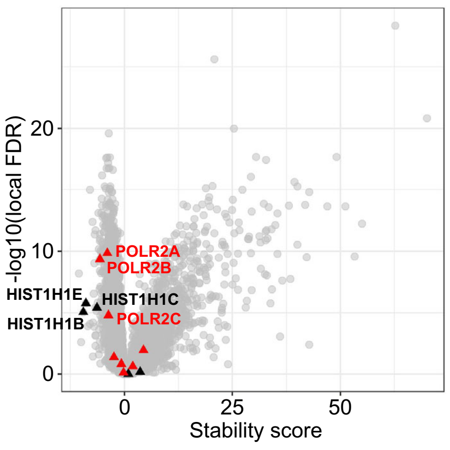

Figure EV5. Stability changes of histone H1 variants and DNA polymerase II proteins.

Volcano plot for stability score. Histone H1 (black triangles) and DNA polymerase II (red triangles) proteins are highlighted. Destabilized proteins are labeled.

Source data are available online for this figure.
